# Effects of table tennis multi-ball training on dynamic posture control

**DOI:** 10.7717/peerj.6262

**Published:** 2019-01-16

**Authors:** Yaodong Gu, Changxiao Yu, Shirui Shao, Julien S. Baker

**Affiliations:** 1 Faculty of Sports Science, Ningbo University, Ningbo, China; 2 School of Science and Sport, University of the West of Scotland, Hamilton, United Kingdom

**Keywords:** Center of pressure, Star excursion balance test, Reach distance, Borg rating of perceived exertion, Musculoskeletal

## Abstract

**Background:**

Prior to the 2017 table tennis season, each participant performed the anterior, posteromedial, and posterolateral the star excursion balance test (SEBT) reach distances in a randomized order. The aim of this study was to assess the effects of table tennis multi-ball training and dynamic balance on performance measures of the SEBT for the male and female.

**Methods:**

The limb lengths of the 12 table tennis athletes were measured bilaterally in the study. Besides warm-up end, the data of this study were recorded at a regular interval at approximately 16 min for the entire multi-ball training session, and they were defined as Phase I, Phase II, Phase II, respectively. The Borg rating of perceived exertion (RPE) scale was used to document the degree of physical strain.

**Results:**

Reaching distances showed a decrease with training progression in all directions. Compared with the male table tennis athletes, the females showed poorer dynamic posture control, particularly when the free limb was considered with the right-leg stance toward posterolateral and posteromedial directions in phase I.

**Discussion:**

This study suggests that during table tennis multi-ball training the male should have a regulatory protocol to compensate the deficit observed in phase II, but the females should be given the protocol in phase I.

## Introduction

Table tennis is one of the most popular sports in the world. According to a report by the International Sports Federation, the population of table tennis participants has reached over 300 million worldwide (Zhang, 2017). Playing table tennis is regarded as a pro-health sporting pastime, which is generally accepted by more and more people who engage in physical activity (Biernat, Buchholtz & Krzepota, 2018). As a rational movement, the characteristics of table tennis play, which involve complex spatial movements of the body that include, acceleration, deceleration, direction change, moving quickly and balance all help players generate optimum stroke production (Girard & Millet, 2009). Table tennis coaches have observed the importance of conditioning and have been proactive in helping table tennis players achieve a better competitive condition. To be successful in the sport, table tennis players are usually asked to perform high intensity training in the preseason as part of their physiological preparation. In addition, technical practice is essential for table tennis players in training sessions, especially during the preseason phase. In order to develop the so-called dynamic stereotype, the table tennis player should try to improve the skills involved when stroking the ball that is in a fixed position or with tactical variations. As a common training method for technical practice, multi-ball training requires the players to repeatedly stroke and return the balls with a combination of feasible footwork (Zhang, 2017), which is generally applied in training sections.

To our knowledge, dynamic balance and fatigue are related (Gioftsidou et al., 2011), and the greater body control for individuals would possess a lower risk of injuries during dynamic movements (Kollock et al., 2018; Knapik et al., 2015). The star excursion balance test (SEBT) is a reliable, valid and easy method to measure lower-limb function in sports and clinical practice (Munro & Herrington, 2010; Hertel, Miller & Denegar, 2000; Kinzey & Armstrong, 1998; Gribble & Hertel, 2003; Bouillon & Baker, 2011). The intra class correlation coefficient values of the SEBT have ranged from 0.67 to 0.96, with high intra rater reliability values of 0.81 to 0.93 (Hertel, Miller & Denegar, 2000; Kinzey & Armstrong, 1998). Therefore, the SEBT has wide practical application in assessing the ability to maintain balance in a single-leg stance in exercise testing, rehabilitation and training (Winter, Patla & Frank, 1990; Robinson & Gribble, 2008). For example, the test has not only been used previously to predict lower limb injury in high school soccer (Bressel et al., 2007; Filipa et al., 2010; Rasool & George, 2007) and basketball (Bressel et al., 2007; Plisky et al., 2006), but also used for convalescent patients with chronic ankle instability (Olmsted et al., 2002; Gribble et al., 2004; Hertel et al., 2006; Hubbard et al., 2007; Isles et al., 2004) and anterior cruciate ligament injuries (Herrington et al., 2009). In addition, it has been applied to evaluate the effects of patellar taping on lower-limb kinematics and dynamic postural control (Aminaka & Gribble, 2008). As a closed-kinetic chain exercise, the SEBT mimics the single-leg squat exercise while attempting maximal reach with the opposite leg, which requires better neuromuscular control, flexibility, balance and strength for the stance leg (Olmsted et al., 2002). Indeed, the SEBT was not only used as a method of assessment, but also as an approach used to improve the performance of movement skills in an athletic population (Munro & Herrington, 2010; Filipa et al., 2010). In addition, a previous study showed that males have larger reach distance when compared with females (Gribble, Hertel & Piegaro, 2003). However, there was no significant gender differences observed when the reach distances were normalized (Gribble et al., 2009). In addition, Day and colleagues (2004) corroborated that the rating of perceived exertion (RPE) is a reliable method to quantify exercise intensities during high-intensity, moderate-intensity and low-intensity resistance training. Faulkner, Parfitt & Eston (2008) found a relationship between the RPE scales and the exercise time during competitive running races. Borg (1982) showed that the scale values, range from 6 to 20 can be used to denote heart rates and cardiovascular responses to exercise (60–200 beats min^−1^). RPE cooperating with SEBT measures the effect of table tennis multi-ball training on dynamic posture control from subjectivity to objectivity for this study.

Dynamic postural-control tasks require contributions from the kinetic chain to generate a greater coordinated movement pattern (Winter, Patla & Frank, 1990; Gribble et al., 2009). As a source of energy, the lower limb can transfer energy upwards to the upper limb through the kinetic chain (Elliott, 2006; Qian et al., 2016). Previous studies reported that lower limb drive was the origin of the kinetic chain, which not only impacts on the quality of skills and tactics used in table tennis (Qian et al., 2016; Fu et al., 2016), but also in lawn tennis (Elliott, 2006; Girard, Micallef & Millet, 2005). These studies showed that power lower-limb drive has an important relationship with optimizing energy transfer in the kinetic chain, which influence the lower-limb flexibility and tactical application. In order to hit back balls from different directions, table tennis players need to be able to adjust their body into a apposite position that facilitates ball return. Moreover, as a multiple-set sport, table tennis players should be able to distribute their energy demand in a way that facilitates continuous strokes and good movement patterns during competition and training. Due to the onset of fatigue, there is a negative relationship between sport performance and playing time (Yaggie & Armstrong, 2004; Pau, Ibba & Attene, 2014). It is well known that the movement of the center of pressure (COP) is a practical parameter used to assess lower extremity performance. Yu et al. (2018) analyzed the trends of load transmission on the foot between squat and standing serves in female table tennis athletes, according to the investigations of COP trajectory. They found squat serve needs higher lower-limb drive compared with standing serve during a short serve. In fact, potential mechanisms that limit performance have been reported, as being related to increasing muscle-spindle discharge that influence the feedback to the central nervous system and then decrease postural control (Gribble et al., 2009; Rozzi et al., 2000; Khin-Myo-Hla, Sakane & Hayashi, 1999). However, to date no study has looked at multi-ball training associated with SEBT and examined what percentage change reflects the degree of exercise intensity that would impact on the performance of table tennis preparation preseason. In addition, there is no study that report the effects of neuromuscular fatigue on the performance of the dynamic postural-control task during table tennis multi-ball training. The aims of the current study are firstly, to identify any relationship between multi-ball training and dynamic postural control in table tennis players; secondly, to provide a guide to formulate feasible pacing strategy for table tennis coaches and players. The research hypotheses of the study were that (1) the normalized reach distance would show decrease when both of the groups perform the SEBT with continuous multi-ball training, and the significant decrease in reach distance would occur later in the male table tennis players compared with the female players, (2) compared with warm-up end, the reach distance for both the dominant and non-dominant leg in the male and female would show different conditions with the training continuing and (3) as training continues, the COP motion (medial-lateral direction, length-X; anterior-posterior direction, length-Y) would show significant changes compared with warm-up end.

## Methods

### Participants

Twelve experts (6 males: age 21.6 ± 1.42 years, weight 73.75 ± 3.24 kg, height 1.78 ± 0.04 m, training experience 14.2 ± 1.46 years; 6 females: age 21.6 ± 1.53 years, weight 63.72 ± 5.4 kg, height 1.65 ± 0.05 m, training experience 14.2 ± 1.62 years) from Ningbo University table tennis team volunteered to participate in the study. All participants were National Division I players, they were right-handed and free from any previous lower limb injuries, surgery, foot diseases and had no previous injury for at least six months. The Ethics Committee of Ningbo University (RAGH20170819) has approved this study, and written informed consent was obtained from all individuals prior to participation. No participant received any payment for this study.

In this study, dominant lower limb was determined according to the ball-kick test (Zakas, 2006). The participants were asked to kick football with arbitrary power and maximal accuracy through a set of obstacles placed 1 m apart and 10 m from the participants, the limb used to kick the football was regarded as the dominant limb and the other side was non-dominant limb.

**Figure 1:**
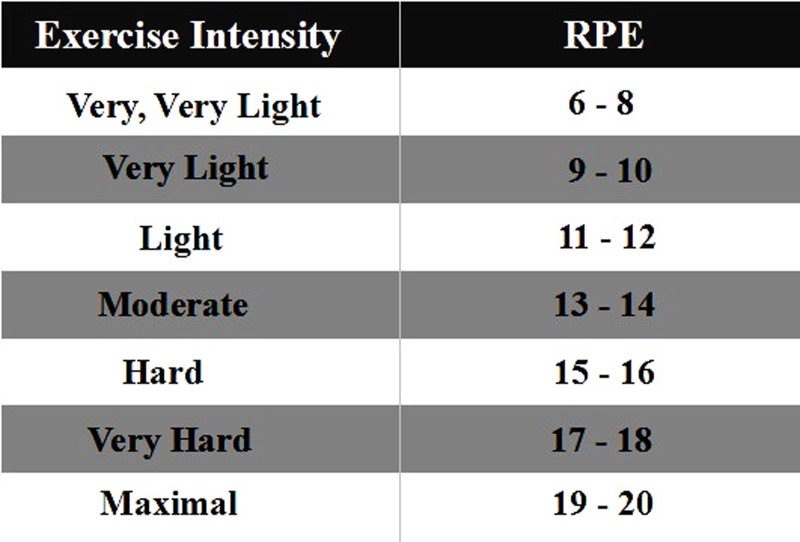
Participants undertook the test procedures consisting of warm-up (W), rest (R), different training phases (Phase I, Phase II, Phase III) and test (T), and end of test (E).

### Procedures

**Figure 2:**
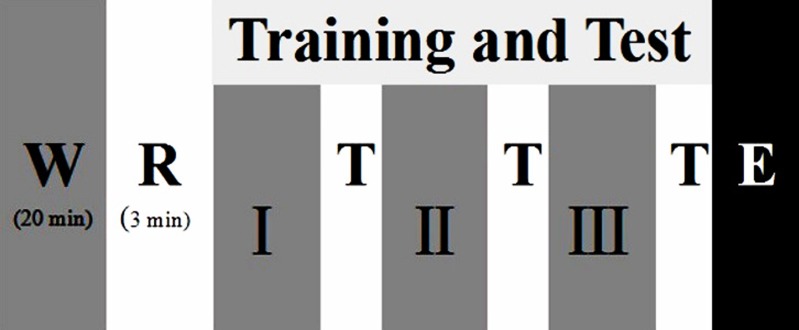
The RPE scale for the study.

This test was executed at Ningbo University table tennis training gymnasium. The study was divided into three sessions, as shown in Fig. 1, each session comprised four phases (warm-up end, Phase I, Phase II, Phase III). The time interval of two sessions for this trial was over 24 h. The first two sessions consisted of a familiarization session that included an instructional video, informed-consent the test procedures, introduction on the Borg 6-20 RPE scale and explanation of how to use the scale. At the third session, each participant was asked to rate his/her perceived exertion based on the RPE scale by answering the question “How was your workout?”: the scale is shown in Fig. 2.

**Figure 3:**
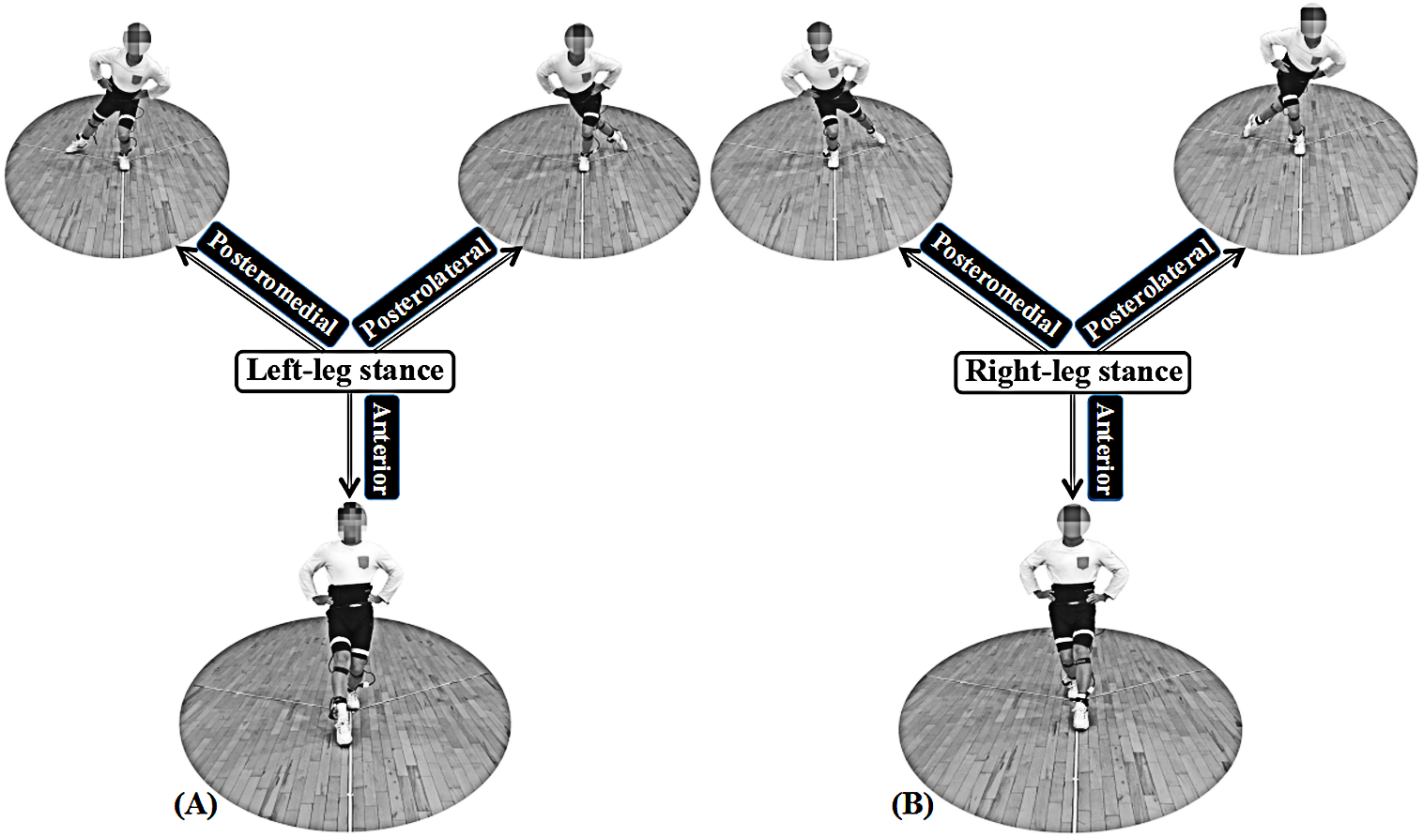
Reaching directions on the SEBT. Note: (A) and (B) show the performance for left- and right-leg stances, respectively.

Before the trials, each participant was given a standardized warm-up of 20 min in the experimental environment. Participants were then assigned multi-ball training protocols in pairs, which included change of direction at impact cooperating topspin, backspin etc. All data sets from the study were recorded at each phase (each phase lasted approximately 16 min) for one entire training session (Malliou et al., 2008). Participants performed the SEBT in the four different training conditions (warm-up end, Phase I, Phase II, Phase III), respectively. Participants were asked to single-leg stand in the center of a grid, then using the free limb to reach in the anterior, posteromedial and posterolateral directions touching lightly—so as not to aid balance—and then return the starting position. The time of one entire session was approximately 50 min. The reach distance (with the big toe as the dependent measure for participants) was recorded when the players could maintain not less than 30 s in each direction. The test was performed from the left leg support to the right leg, and both lower limbs were measured under the same conditions respectively. Each participant performed eight times in each direction. The mean of the top five reach distances for each participant in the three reach directions were used for further data analysis (Fig. 3).

At the initial session, to calculate the dependent variable for normalized reach distance, leg length was measured with the participants in supine position. The participants’ right and left leg lengths were measured (respectively), from the anterior superior iliac to the distal end of the medical malleolus with a calibrated tape measure.

The trial was discarded and repeated when: (1) the stance leg lost balance, (2) the heel of the stance leg lost contact, (3) the support time was less than required, and (4) the reaching foot could not return the starting position.

### Instrumentation

COP was recorded by a Novel Pedar insole plantar pressure measurement system (Novel GmbH, Munich, Germany) at a frequency of 50 Hz. This equipment has been previously used in kinetic analysis for table tennis and tennis (Girard et al., 2010; Fu et al., 2016; Qian et al., 2016). The length of COP motion includes length-X and length-Y, which are routinely used to assess postural control (Fu et al., 2016). Measuring insoles were placed bilaterally inside the participants’ shoes (size 38–42 cm), and the data recording was sampled through Bluetooth technical equipment. This equipment did not influence technical motions, and was worn in both the training times and the trial processes, respectively.

### Statistical analysis

All statistical tests were performed using SPSS version 19.0 software (SPSS Inc., Chicago, IL, USA) for Windows. Prior to statistical comparisons, an initial Shapiro–Wilks test confirmed that all data were normally distributed. Descriptive statistics were used to calculate the means ±  standard deviations for all participants. To examine the differences in the reach distance of dominant and non-dominant limbs during the SEBT for the male or the female, independent *t*-tests were carried out, respectively. There were pairwise comparisons in two different phases for the RPE, reach distance and length of COP (-X and -Y), one-way repeated-measures analysis of variance (ANOVA) was used to determine differences in the four training stages. The significance level for all tests was set at *p* ≤ 0.05. The effect size was determined based on Cohen’s d which was used to compare the differences in the average of the two groups. Effect size (ES) is evaluated as trivial (≥ 0.19), small (≥ 0.2 and ≤ 0.49), medium (≥ 0.50 and ≤ 0.79) and large (≥ 0.80), respectively (Cohen, 1992).

## Results

### RPE

**Figure 4:**
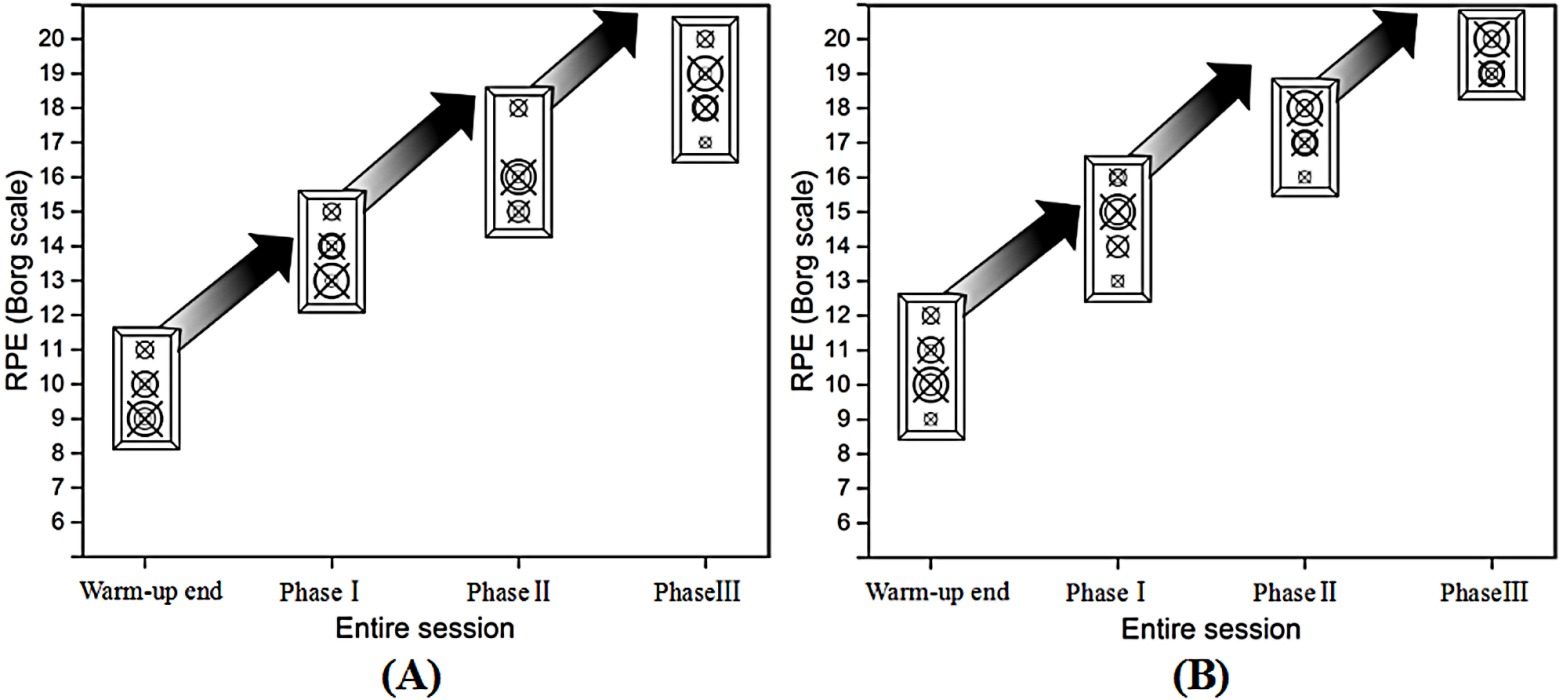
Trend of RPE for the male (A) and female (B) during the multi-ball training in each phase.

Descriptive characteristics of the participants in the four phases are presented in Table 1. As expected, significant differences were found in phase I, phase II and phase III compared with warm-up (Fig. 4).

**Table 1:** Mean  ±  standard deviations (mean  ±  SD), standard error of measurement (SEM), 95% confidence intervals (CI), effect sizes (ES) for the RPE values at warm-up end, phase I, phase II and phase III.

**Phase**	**Gender**	**Mean ± SD**	**SEM**	**CI**	**ES**
**Warm-up end**	Male	9.67 ± 0.82	0.33	(8.81, 10.52)	–
	Female	10.50 ± 1.05	0.43	(9.40, 11.60)	–
**Phase I**	Male	13.83 ± 0.75	0.31	(13.04, 14.62)	^a^ 0.93
	Female	14.83 ± 1.17	0.48	(13.61, 16.06)	^a^ 0.89
**Phase II**	Male	16.00 ± 1.10	0.45	(14.85, 17.15)	^a^ 0.95 ^b^ 0.75
	Female	17.17 ± 0.75	0.31	(16.38, 17.96)	^a^ 0.96 ^b^ 0.77
**Phase III**	Male	18.50 ± 1.05	0.43	(17.40, 19.60)	^a^ 0.97 ^b^ 0.93 ^c^ 0.75
	Female	19.33 ± 0.52	0.21	(18.79, 19.88)	^a^ 0.98 ^b^ 0.92 ^c^ 0.86

**Notes.**

a Shows a comparison with warm-up end (*p* ≤ 0.05).

b Shows a comparison with phase I (*p* ≤ 0.05).

c Shows a comparison with phase II (*p* ≤ 0.05).

### SEBT

**Figure 5:**
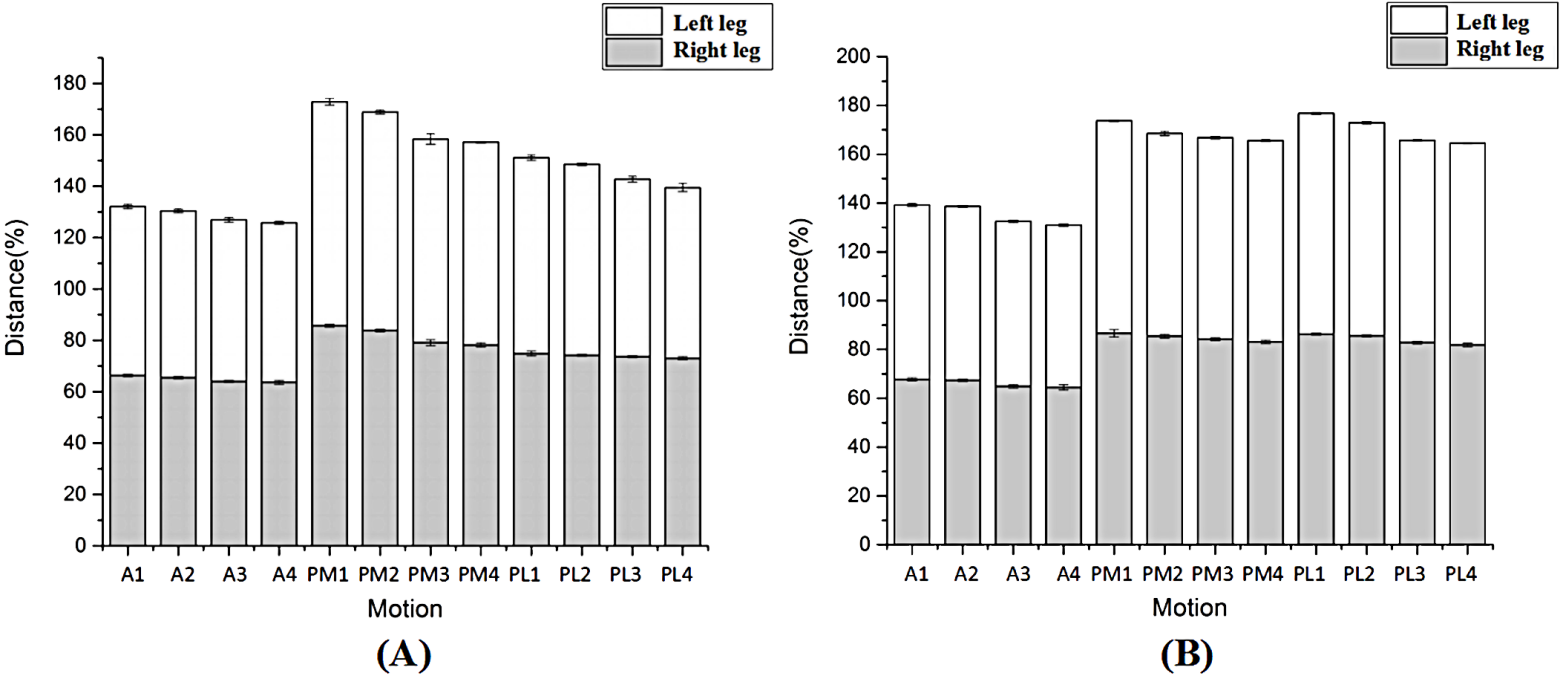
The comparison of entire trend in the male (A) and female (B)

As Fig. 5 shown, there were similar high between left and right legs in reaching distances when performed the SEBT for the two groups. Based on independent t-tests, the reaching distance had no significant differences in the three reach directions of the each phase between dominant and non-dominant limbs for the male or female (*p* > 0.05) (Fig. 5). In addition, compared with warm-up, the reach distance of the right leg for the male participants at phaseII showed significant decrease in the anterior direction and posteromedial direction (Tables 2, 3). The reach distance of the left leg for the male participants at the warm-up end showed significantly greater distance in the posterolateral direction and posteromedial direction than phase II and III, in addition, greater distances were recorded in the anterior direction compared with the phase III (Tables 2 and 3). For the female participants, there were no significant changes in the all reach distances observed for the right leg except the posterolateral direction at phase II and III when compared with warm-up end (Tables 2 and 4). However, for the reach distance of the left leg, there were significant differences in all directions except the anterior direction at phase I when compared with warm-up (Tables 2 and 4).

**Table 2:** Distance on the SEBT for each direction (mean  ±  SD).

**Direction/ Limb/Group**		**Warm-up end**	**Phase I**	**Phase II**	**Phase III**
Anterior					
Right					
	Male	66.33 ± 0.59	65.60 ± 0.58	64.81 ± 1.06^a^	64.00 ± 1.14^a^^,^^b^
	Female	67.74 ± 0.78	67.33 ± 0.94	66.95 ± 0.60	66.87 ± 0.98
Left					
	Male	65.81 ± 1.09	65.11 ± 0.34	64.95 ± 0.84	64.30 ± 0.73^a^
	Female	71.73 ± 0.71	71.13 ± 0.60	67.20 ± 0.77^A^^,^^B^	66.37 ± 0.79^A^^,^^B^
Posteromedial					
Right					
	Male	85.40 ± 0.87	84.40 ± 0.59	79.13 ± 1.44^a^^,^^b^	78.21 ± 1.02^a^^,^^b^
	Female	86.43 ± 1.76	85.43 ± 1.30	85.17 ± 1.01	85.05 ± 1.70
Left					
	Male	86.77 ± 1.49	85.26 ± 1.12	78.97 ± 2.60^a^^,^^b^	78.91 ± 0.39^a^^,^^b^
	Female	87.00 ± 0.47	82.96 ± 0.74^A^	82.60 ± 0.37^A^	82.52 ± 0.23^A^
Posterolateral					
Right					
	Male	75.05 ± 1.09	74.88 ± 0.82	74.42 ± 0.86	74.14 ± 0.87
	Female	86.11 ± 0.43	85.63 ± 0.54	82.93 ± 0.79^A^^,^^B^	82.12 ± 1.18^A^^,^^B^
Left					
	Male	75.44 ± 1.40	74.91 ± 0.77	68.18 ± 1.65^a^^,^^b^	67.49 ± 2.14^a^^,^^b^
	Female	90.54 ± 0.32	87.16 ± 1.08^A^	83.14 ± 0.63^A^^,^^B^	82.78 ± 0.53^A^^,^^B^

**Notes.**

Mean  ±  standard deviations are normalised reach distance (reach distance/leg length ×100).

a and A Significant difference from warm-up end in male and female (*p* ≤ 0.05), respectively.

b and B Significant difference from phase I in male and female (*p* ≤ 0.05), respectively.

c and C Significant difference from phase II in male and female (*p* ≤ 0.05), respectively.

**Table 3:** Standard error of measurement (SEM), 95% confidence intervals (CI), effect sizes (ES) for trials of males in each direction.

		**Anterior**		**Posteromedial**		**Posterolateral**	
		Right	Left	Right	Left	Right	Left
**Warm-up end**	SEM	0.24	0.44	0.36	0.61	0.45	0.57
	CI	(65.72, 66.94)	(64.67, 66.95)	(84.49, 86.32)	(85.21, 88.34)	(73.90, 76.20)	(73.97, 76.90)
**Phase I**	SEM	0.24	0.38	0.24	0.45	0.34	0.31
	CI	(64.98, 66.21)	(64.12, 66.09)	(83.79, 85.02)	(84.10, 86.43)	(74.02, 75.74)	(74.11, 75.72)
	ES(95% CL)	^a^ 0.53	^a^ 0.40	^a^ 0.56	^a^ 0.50	^a^ 0.09	^a^ 0.23
**Phase II**	SEM	0.43	0.34	0.59	1.06	0.35	0.67
	CI	(63.70, 65.92)	(64.07, 65.82)	(77.61, 80.64)	(76.23, 81.70)	(73.51, 75.33)	(66.45, 69.90)
	ES(95% CL)	^a^ 0.66 ^b^ 0.42	^a^ 0.40 ^b^ 0.12	^a^ 0.93 ^b^ 0.92	^a^ 0.88 ^b^ 0.84	^a^ 0.31 ^b^ 0.26	^a^ 0.92 ^b^ 0.93
**Phase III**	SEM	0.47	0.30	0.41	0.16	0.35	0.87
	CI	(62.80, 65.20)	(63.54, 65.06)	(77.15, 79.26)	(78.50, 79.32)	(73.23, 75.05)	(65.25, 69.73)
	ES(95% CL)	^a^ 0.79 ^b^ 0.66 ^c^ 0.35	^a^ 0.63 ^b^ 0.58 ^c^ 0.38	^a^ 0.97 ^b^ 0.97 ^c^ 0.35	^a^ 0.96 ^b^ 0.97 ^c^ 0.02	^a^ 0.42 ^b^ 0.40 ^c^ 0.16	^a^ 0.91 ^b^ 0.92 ^c^ 0.18

**Notes.**

All values are normalised reach distance (reach distance/leg length ×100).

a Shows a comparison with warm-up end (*p* ≤ 0.05).

b Shows a comparison with phase I (*p* ≤ 0.05).

c Shows a comparison with phase II (*p* ≤ 0.05).

**Table 4:** Standard error of measurement (SEM), 95% confidence intervals (CI), effect sizes (ES) for trials of females in each direction.

		**Anterior**		**Posteromedial**		**Posterolateral**	
		Right	Left	Right	Left	Right	Left
**Warm-up end**	SEM	0.32	0.29	0.72	0.20	0.18	0.13
	CI	(66.92, 68.57)	(70.99, 72.47)	(84.58, 88.28)	(86.51, 87.50)	(85.66, 86.56)	(90.20, 90.88)
**Phase I**	SEM	0.38	0.24	0.53	0.30	0.22	0.44
	CI	(66.34, 68.31)	(70.51, 71.75)	(84.07, 86.79)	(82.18, 83.73)	(85.07, 86.19)	(86.03, 88.30)
	ES(95% CL)	^a^ 0.23	^a^ 0.42	^a^ 0.31	^a^ 0.96	^a^ 0.44	^a^ 0.90
**Phase II**	SEM	0.24	0.31	0.41	0.15	0.32	0.26
	CI	(66.32, 67.57)	(66.40, 68.01)	(84.12, 86.22)	(82.21, 82.99)	(82.10, 83.76)	(82.47, 83.80)
	ES(95% CL)	^a^ 0.49 ^b^ 0.23	^a^ 0.95 ^b^ 0.94	^a^ 0.40 ^b^ 0.11	^a^ 0.98 ^b^ 0.29	^a^ 0.93 ^b^ 0.89	^a^ 0.99 ^b^ 0.92
**Phase III**	SEM	0.40	0.32	0.70	0.95	0.48	0.22
	CI	(65.84, 67.89)	(65.54, 67.20)	(83.26, 86.84)	(82.28, 82.76)	(80.88, 83.35)	(82.22, 83.33)
	ES(95% CL)	^a^ 0.44 ^b^ 0.23 ^c^ 0.05	^a^ 0.96 ^b^ 0.96 ^c^ 0.47	^a^ 0.37 ^b^ 0.12 ^c^ 0.04	^a^ 0.99 ^b^ 0.37 ^c^ 0.13	^a^ 0.91 ^b^ 0.89 ^c^ 0.37	^a^ 0.99 ^b^ 0.93 ^c^ 0.30

**Notes.**

All values are normalised reach distance (reach distance/leg length ×100).

a Shows a comparison with warm-up end (*p* ≤ 0.05).

b Shows a comparison with phase I (*p* ≤ 0.05).

c Shows a comparison with phase II (*p* ≤ 0.05).

### COP

**Figure 6:**
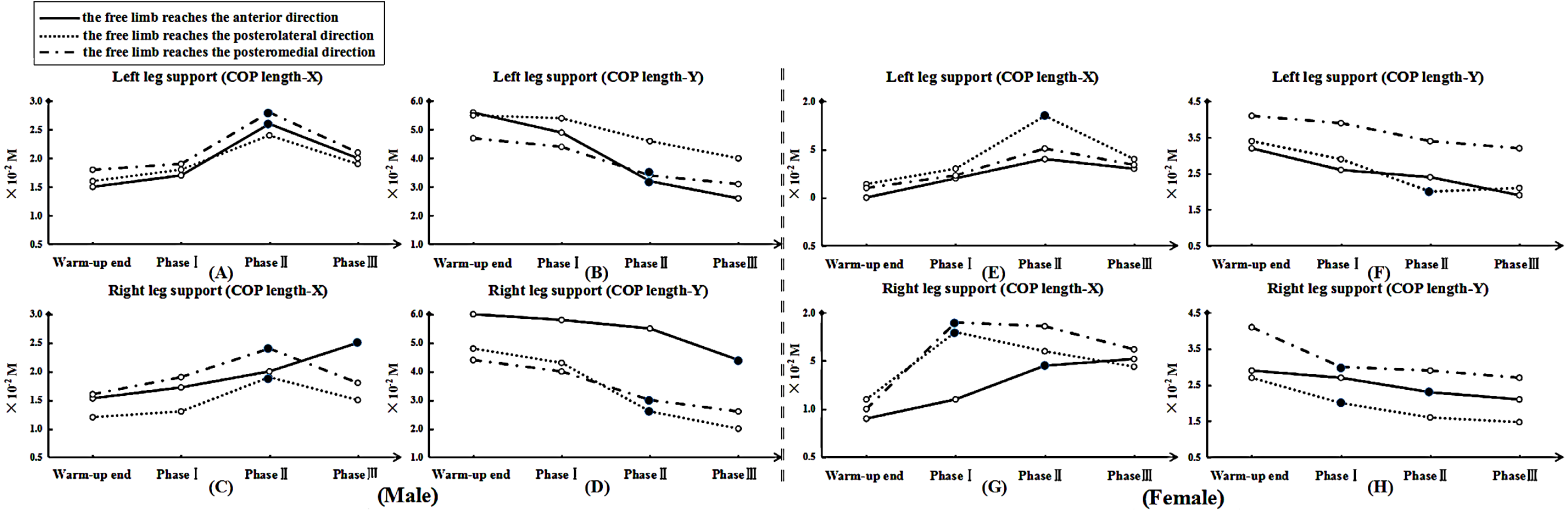
The length of COP motion in each phase. Note: “.” means significant difference began to show in COP length-X or -Y compared with warm-up end. For male, (A), (B), (C) and (D) show the length of COP motion (X- or Y- axis) at left and right leg support, respectively. For female, (E), (F), (G) and (H) show the length of COP motion (X- or Y- axis) at left and right leg support, respectively.

Figure 6 displays the length of COP in each phase. For all participants, length-X increased more than warm-up end, but length-Y decreased more compared with warm-up end following continuous training when performed the SEBT in all directions (*p* ≤ 0.05). For the male participants, when the right lower limb reached anterior and posteromedial directions, length-X of the support leg in phase II was significantly greater, but length-Y was significantly smaller than warm-up end (*p* ≤ 0.05). Similarly, compared with warm-up end, length-X of the right leg was significantly greater in phase II and III when the free limb reached posteromedial/posterolateral and anterior directions (respectively), but length-Y was significantly smaller. For female participants, the length-X of the left leg was significantly greater in phase II than warm-up end when the free limb reached posterolateral direction, but length-Y was significantly smaller in the same phase. In addition, compared with warm-up end, length-X of the right leg was significantly greater. The free leg reached the anterior direction in phase II and it reached the posteromedial/posterolateral direction in phase I, but length-Y were significantly smaller.

## Discussion

To our knowledge, this is the first study that has provided evidence related to the relationship between dynamic balance and multi-ball training in preseason table tennis. The purpose of this study was to assess the effects of multi-ball training on dynamic posture control using the SEBT during table tennis multi-ball training. Based on the findings of the study, the results and analysis could help coaches to better regulate the training pace during preseason preparation. As expected, the results of the study indicated that the significant decrease in dynamic balance mainly occurred in phase II, but the female participants started to decline earlier than the males. Additionally, when postural control started to decrease significantly, length-X of COP motion showed to increase more than that observed at the warm-up end. The length-Y of COP motion showed significant reductions when the same stages were compared.

It is well documented that the Borg RPE scale has become a standard method to evaluate perceived exertion and has been used in exercise science to quantify exercise intensity (Day et al., 2004; Noble et al., 1983; Noble & Robertson, 1996). In addition, Foster et al. (1996); Foster (1998); Foster et al. (2001); Foster, Rodriguez-Marroyo & De Koning (2017) studied the specific stages of exercises in an entire aerobic exercise session using the RPE scale. They provided evidence that this method can be applied to quantifying the intensity of exercise during various types of exercise. The results of our study found that there were apparently different training loads between each phase for both the male and female participants. This provided useful information for comparative purposes between the male and female participants. Considering the characteristics of table tennis multi-ball training, this finding may be related to the training time observed and the duration of the activity may be contributing to the fatigue profiles recorded. However, because we only used one method it may be speculative to try and explain the relationship between dynamic balance and training time. Therefore, further work is needed to examine in detail any relationship observed.

Plisky et al. (2006) have indicated that potential risk factors for injury may result in the opposite limb if a reduced reach distance in the free limb is observed when using the measurement method employed in the SEBT. Previous studies have indicated that the reduced work of skeletal muscle is associated with muscle fatigue, which was believed to be a potential cause of increased injury rates for athletes during unexpected perturbations (Hassanlouei et al., 2012). Additionally, in racket sport players, there is a potential source of muscle asymmetry between two legs, which could largely affect the pattern of movement and perhaps induce sport injuries (Ye, Sun & Fekete, 2018; Lam et al., 2018; Ireland et al., 2013; Sanchis-Moysi et al., 2010). The less adept lower limb for balance, the greater the inability to provide an optimally stable base for the neuromuscular system to maintain a constant tension. This could promote absorption of increased load and contribute to the instability recorded (Fang, 2018; Plisky et al., 2006; Hewett, Myer & Ford, 2001; Weaver et al., 1999). In addition, Earl & Hertel (2001) showed that the support leg muscles could be activated to a different extent during the SEBT, which could be contributing to postural sway under during continuous table tennis multi-ball training. Exercise-induced fatigue would result in failure to produce maximal force and reduce flexibility, which could influence motor performance. Previous studies have corroborated that fatigue-induced impairments could badly impact on joint proprioception and neuromuscular control (Hassanlouei et al., 2012; Voight et al., 1996; Ribeiro et al., 2008; Miura et al., 2004). Boden et al. (2000) reported that these findings constantly occurred at the end stages of athletic competition. Additionally, the motions of sudden deceleration, landing and pivoting maneuvers throughout the entire table tennis multi-ball training regime would exacerbate the risk of sport injury. In order to try and quantify the influence of postural control on a participant performance, Caron et al. (2000) measured the displacement of the COP. The findings from our study in relation to COP displacement indicated that the reach distance in nearly all directions was beginning to show a sharp decline trend in phase II. At the same time, in order to maintain posture balance, the length-X of the COP showed increases, but its length-Y showed a decrease when compared with the warm-up end. The results indicated that dynamic balance was significantly decreased with table tennis multi-ball training. When we consider the step features in table tennis, that include suddenly stopping and pivoting, which place repeated rotational shear and loading forces on each joint of the lower limbs during table tennis multi-ball training, it may be useful to compare the actions with tennis players. Tennis players are at risk of increasing overuse and acute injuries including ligament sprains, chronic muscle strain, hamstring strains, stress fractures, ankle sprains, shin splints, knee contusions and growth plate injuries (Murphy, Connolly & Beynnon, 2003; Kibler & Safran, 2005; Hutchinson et al., 1995). According to the report of Kondrič et al. (2011), they found that the most frequent injuries in racket sports is muscle tissues from training and/or competition processes, and there is a high percentage of injuries in lower-limb joints (ankle and foot in particular; 23.69% in total). They also indicated that due to the characteristics of abrupt blocking movements in playing table tennis, the percentage of hip injuries exists 5.76%. In relation to these findings since table tennis players exhibited a apparent decline in dynamic balance in phase II, a sport injury prevention program may be beneficial.

As a method of neuromuscular training, the SEBT can be incorporated into a pre-participation physical examination for athletes to improve identified specific deficits in the preseason phase of competition (Plisky et al., 2006). Paterno et al. (2004) and Holm et al. (2004), reported that a period of 6–7 weeks of balance training would help athletes rapidly improve postural stability. Based on the finding of this study, these methods should be incorporated into preseason training and preparation phases. Future work would include training monitoring being dominated by emerging new technologies that would consist of real-time monitoring of the internal and external forces during training and recovery (Foster, Rodriguez-Marroyo & De Koning, 2017). Therefore, the RPE and the SEBT may be merged to formulate exciting training monitoring design via new and exciting technologies.

Some limitations to this study should be noted. Firstly, the data were based on one table tennis season using one university table tennis team. Although all participants were granted with National DivisionI status, it may limit the external validity at some degree. Further, the study did not collate lower-limb strength and flexibility data. There is another potential limitation is that we did not have electromyographic data to analyze the internal mechanism of inducing decrease in dynamic balance from the standpoint of neuromuscular system. In addition, future studies should consider research during competition to assess the relationship between dynamic balance, strength and time duration.

## Conclusions

This study examined the relationship between dynamic posture control and the time of table tennis multi-ball training preseason. The study’s prospective design allowed the requirements of each trainee to reduce the risk of injury during table tennis multi-ball training. Further, the findings of this study may assist the coaches and the certified athletic trainees to understand the effects of table tennis multi-ball training on dynamic posture control. The findings may also be useful in determining musculoskeletal deficits in performance. Finally, the findings may also help in the design and implementation of specific rehabilitative programs when the postural control descends significantly.

##  Supplemental Information

10.7717/peerj.6262/supp-1 Data S1 Raw data exported from the rating of perceived exertion (RPE) for the male and female Multi-ball training in each phase applied for data analyses and preparation for the detailed investigation shown in Fig. 4 and Table 1.

10.7717/peerj.6262/supp-2 Data S2 Raw data exported from the distance on the star excursion balance test for each direction applied for data analyses and preparation for the detailed investigation shown in Fig. 5 and Tables 2, 3 and 4

10.7717/peerj.6262/supp-3 Data S3 Raw data exported from the length of COP motion in each phase applied for data analyses and preparation for the detailed investigation shown in Fig. 6

## Additional Information and Declarations

### Data Availability

The following information was supplied regarding data availability: The raw data are provided in the Supplemental Files.
